# Abiotic selection of microbial genome size in the global ocean

**DOI:** 10.1038/s41467-023-36988-x

**Published:** 2023-03-13

**Authors:** David K. Ngugi, Silvia G. Acinas, Pablo Sánchez, Josep M. Gasol, Susana Agusti, David M. Karl, Carlos M. Duarte

**Affiliations:** 1 Leibniz Institute DSMZ – German Collection of Microorganisms and Cell Cultures, Braunschweig, Germany; 2 Department of Marine Biology and Oceanography, Institut de Ciències del Mar, CSIC, Barcelona, Spain; 3 King Abdullah University of Science and Technology, Red Sea Research Center, Thuwal, Saudi Arabia; 4 Department of Oceanography, School of Ocean and Earth Science and Technology, University of Hawaií at Mãnoa, Honolulu, USA

**Keywords:** Microbial ecology, Molecular ecology, Metagenomics

## Abstract

Strong purifying selection is considered a major evolutionary force behind small microbial genomes in the resource-poor photic ocean. However, very little is currently known about how the size of prokaryotic genomes evolves in the global ocean and whether patterns reflect shifts in resource availability in the epipelagic and relatively stable deep-sea environmental conditions. Using 364 marine microbial metagenomes, we investigate how the average genome size of uncultured planktonic prokaryotes varies across the tropical and polar oceans to the hadal realm. We find that genome size is highest in the perennially cold polar ocean, reflecting elongation of coding genes and gene dosage effects due to duplications in the interior ocean microbiome. Moreover, the rate of change in genome size due to temperature is 16-fold higher than with depth up to 200 m. Our results demonstrate how environmental factors can influence marine microbial genome size selection and ecological strategies of the microbiome.

## Abstract (web summary)

This study investigates the average genome size of planktonic prokaryotes across tropical and polar oceans and down to the hadal realm. Using hundreds of metagenomes of marine microorganisms, genome size was found to be highest in the perennially cold polar ocean, suggesting that environmental factors influence genome size selection and the ecological strategies of marine microbes.

## Introduction

Genome size is a viable predictor of the metabolic complexity of prokaryotes (bacteria and archaea) and a fundamental force shaping their diversity and niche occupation in Earth’s biomes, including the ocean^1–4^. Our understanding of the mechanisms underlying the evolution of microbial genome size has changed greatly in the last two decades, with multiple evolutionary and ecological processes implicated in shaping genome size and complexity in nature, including streamlining, selection for metabolic efficiency, genetic drift, natural selection, homologous recombination (e.g., via lateral/horizontal gene transfer and mobile genetic elements), and increased mutation rates^1,4–7^. The variation of genome size can therefore be thought as a function of complex evolutionary forces, reflecting the interplay of environmental, biological, and historical factors.

For example, nutrient limitation is considered a strong selective force that causes the relatively low guanine and cytosine content and genome streamlining in pelagic bacterioplankton^1,3,4,8,9^. The abundant cyanobacterium *Prochlorococcus* and the heterotrophic alphaproteobacterium *Pelagibacter* (SAR11), which are prevalent in the tropical epipelagic ocean, are examples of this model^10^. However, several recent studies argue against widespread streamlining, and instead advocate genetic drift as the dominant force compacting prokaryotic genomes^7,10,11^. In parallel, mathematical models suggest that greater variability of an environmental selection pressure leads to genome enlargement due to an increase in gene copy number^12^. Genome size expansion is also attributed to increased mortality, gene family size expansion, and horizontal gene transfer common in ecosystems with high environmental pressure and strong predator-prey relationships^12^. Thus, the evolution of prokaryote genome size may be influenced by multiple ecological and evolutionary mechanisms, but a comprehensive understanding of how these processes affect genome evolution in the ocean on a global scale is lacking.

Two candidate environmental variables in the ocean that may affect microbial genome size are ocean depth and temperature. Pioneering studies based on marine isolates and microbial metagenomes have shown that the average genome size (AGS) of planktonic prokaryotes increases with ocean depth^8,13–16^. In these studies, it was speculated that a more relaxed purifying selection favors large genomes at depth compared to the surface, reflecting the greater diversity of organic compounds available in the deep sea and the small population sizes. Across the ensemble of cultured planktonic prokaryotes, genomes are much larger in the nutrient-rich and cold bathypelagic ocean than in the warm oligotrophic epipelagic ocean^14^. However, much of the marine prokaryotic diversity has yet to be cultured^17^, with current estimates suggesting that two to ten million prokaryotic species exist in the global ocean^18,19^. Combined with the fact that the average ocean depth is about 4000 m^20^ and that physicochemical properties of the oceanic water column remain relatively constant at great depth^21^, implies that there may be a million undescribed species and planktonic microorganisms with even larger genomes and biological adaptations (e.g., to cold) inhabiting the unexplored deep-sea habitat.

However, genome sizes of uncultured oceanic prokaryotes have been studied only locally [i.e., North Pacific^8,13^] or only for the photic ocean based on metagenomic sequences^15,16^, leaving open the question of whether previous findings based on limited datasets are applicable at global scales and different oceanic provinces, especially considering the vast collection of metagenomes now available. The impact of eukaryotic and viral genomes sequenced concurrently with prokaryotic metagenomes on AGS prediction also remains unclear, as these factors can bias the accuracy of AGS estimation [see, ref. ^22^], but were not thoroughly considered in previous studies^15,16^.

Despite its important role in shaping the structure and function of the global ocean microbiome^23^, the question of how temperature contributes to the evolution of genome size in marine prokaryotes has not been extensively studied. Understanding genome evolution in the vast expanse of the ocean is important because ocean temperature varies widely, from ~4 °C in the deep ocean interior^20^ to 32 °C in the tropical pelagic ocean [e.g., Red Sea^24^], remains relatively constant in the polar ocean, and is increasing due to the effects of climate change^25^. Also, spontaneous mutation rates increase at high temperatures^26,27^, leading to genome size reduction in prokaryotes^7^, while adaptation to changing environmental conditions causes genome size expansion due to gene duplication^28–30^. Consequently, genome sizes of marine microorganisms in the pelagic and in the deep ocean are expected to evolve independently due to large temperature variations in these depths. This is because the epipelagic features high but variable temperatures and is accompanied by the widespread mutagenic effects of UV-induced DNA damage (see, ref. ^31^), while the interior ocean has relatively constant low temperatures (~4 °C). Indeed, the genome size of some cultured microbes is negatively correlated with the optimal growth temperature of the microbes^32^, but these are microbes from different environments where habitat could also play a selective role. Overall, we hypothesize that smaller genomes would predominate in the upper pelagic ocean, where ambient temperatures approach the growth optimum but increase mutation rates, while relatively larger genomes would predominate in the ‘colder’, nutrient-rich interior of the ocean and in perennially cold polar waters.

Here, we examine massive metagenomic sequence compilations of global ocean microbiomes (Supplementary Data 1), covering environmental gradients^33^, temporal sampling campaigns^8^ and global assessments^34–36^, to elucidate the relationship between estimated average microbial genome size (AGS) and ambient environmental variables in vertically matched metagenomic samples extending to the hadal domain and covering major ocean provinces. Our results confirm previous reports of genome size increases with ocean depth and shed further light on the large magnitude of thermal control on the evolution of genome size of marine microbes relative to depth on a global scale in the tropical and polar oceans.

## Results

### Non-prokaryotic metagenomic sequences confound average genome size estimations

**Fig. 1:**
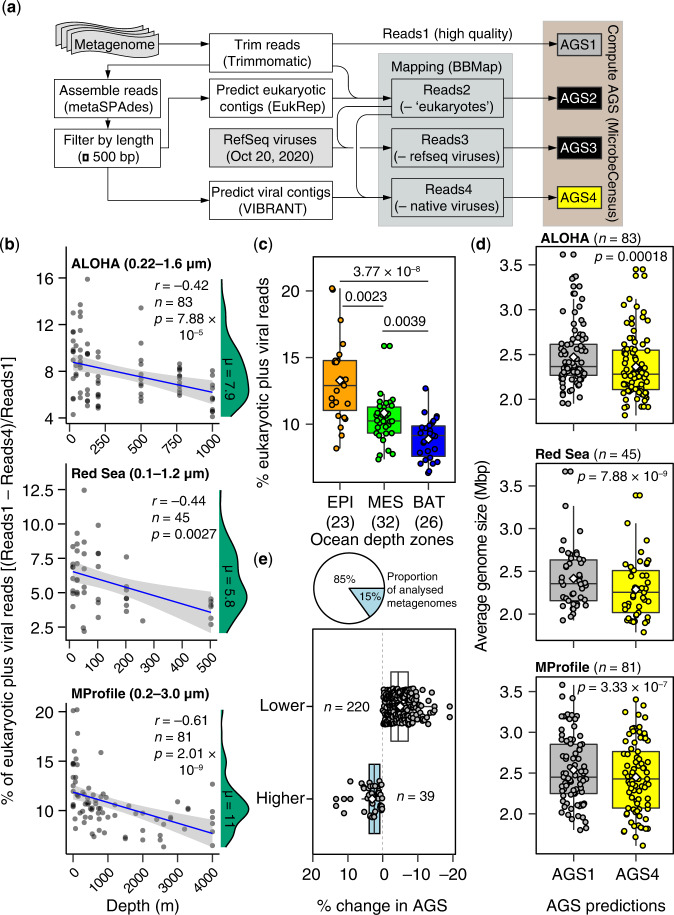
Eukaryotic and viral metagenomic reads bias AGS estimates in marine microbial metagenomes. **a** Schematic workflow of procedures used for estimating AGS in metagenomic samples. AGS is estimated based directly on preprocessed high-quality metagenomic reads (AGS1) and after three iterative steps to remove potential eukaryotic reads (AGS2) and viral reads detected based on the RefSeq viral genome database (AGS3) or de novo (AGS4). See the “Methods” section for more details. **b** Relationship between depth and proportion of total putative eukaryotic and viral sequences in marine metagenomic collections. The blue line indicates the fitted one-tailed Spearman correlation (*r*), with the corresponding 95% confidence intervals for the curve indicated by grey bands. The density distribution of the estimated proportion of contaminants is shown in green, with the corresponding median values (*µ*) highlighted. Values in parenthesis denote the filter size range of sampled metagenomes. **c** The fraction of ‘contaminating’ reads is highest in the epipelagic ocean relative to other ocean depth layers. EPI Epipelagic (~3–200 m), MES Mesopelagic (200–1000 m), BAT Bathypelagic (1000–4000 m). Values in parenthesis indicate the number of metagenomes. Only the results from the Malaspina Vertical Profiles (MProfile) metagenomes are shown as they cover greater depths of the global ocean (mean 1114 m; Supplementary Data 1). **d** Eukaryotic and viral metagenomic sequences significantly increase AGS estimates for prokaryotic plankton in marine metagenomes. Values in parenthesis show number of metagenomes for AGS1 and AGS2. **e** AGS estimates decreased in most metagenomic samples (85%; *n* = 220) after decontamination compared to predictions directly from preprocessed metagenomes by 1–19% (*n* = 39). Boxplots (**c**–**e**) show the median as middle horizontal (**c**, **d**) or vertical (**e**) lines and interquartile ranges as boxes (whiskers extend no further than 1.5 times the interquartile ranges). Data are shown as circular symbols, while mean values are shown as white colored diamonds. Values at the top (**c**, **d**) indicate the adjusted significant *P*-values of the unpaired (**c**) and paired (**d**) two-sided Wilcoxon test with Benjamini-Hochberg correction. Source data are provided as a Source Data file.

In this work, we employed MicrobeCensus^22^ for de novo estimation of the average genome size (AGS) of microorganisms captured in shotgun metagenome sequences (Fig. 1a; Supplementary Data 1). Briefly, MicrobeCensus optimally aligns metagenomic reads to a set of 30 conserved single-copy gene (CSCG) families found in prokaryotes ^22^. Based on these mappings, the relative abundance of each CSCG is then computed and used to estimate AGS based on the proportionality principle—that is, the AGS of the community is inversely proportional to the relative abundance of each marker genes^22^. Finally, a weighted average AGS is calculated that excludes outliers to obtain a robust AGS estimate for a given metagenomic sample^22^.

Of note, the AGS of complete prokaryotic genomes increases with the cumulative number of associated phages and other mobile genetic elements^37^. Similarly, AGS estimates derived from metagenomic sequences of uncultured “free-living” microbes (captured in 0.1–3 µm-size filters) may also be affected by putative phage and eukaryotic microbiomes sequenced concurrently in fractionated seawater samples (see,^8,22^). To evaluate this possibility in our AGS predictions, we compared AGS estimates obtained directly from quality-controlled metagenomes with estimates from the same metagenomes iteratively subjected to three (de novo) decontamination procedures to filter out potential eukaryotic and viral sequence reads (Fig. 1a; see details in the “Methods” section). Overall, putatively ‘contaminating’ viral and eukaryotic reads accounted for 1% to 20% (average 7.5%) of the high-quality trimmed sequences in the four microbial metagenome collections (Fig. 1b; Supplementary Data 1). As expected, the average proportion of contaminating sequences in metagenomes from large (0.2–3.0 µm) and small (0.1–1.2 µm) size fraction filters were the highest (~11%) and lowest (~5%), respectively (Fig. 1b). In addition, the proportion of contaminating reads was significantly dependent on the depth layer of the ocean (Kruskal-Wallis χ^2^ = 32.40, df = 2, *p* < 0.001); the lowest values were in the bathypelagic (Fig. 1c).

Crucially, significant differences were observed in AGS estimates in the presence and absence of contaminating reads (repeated measures ANOVA; *p* < 0.0001; Supplementary Data 2), regardless of the metagenome sample, its geography, or the range of filters used (0.1–3 µm; Fig. 1d). The vast majority of metagenomes (85% of 259 samples) showed AGS estimates that were ~1–19% lower (average 5%) after the putative eukaryotic and viral metagenomic sequences were removed (Fig. 1e). These results suggest that environmental eukaryotic and viral genomic sequences affect AGS predictions for prokaryotes in marine metagenomes. Therefore, the AGS estimates reported and discussed below are based on high-quality metagenomes lacking putative viral and eukaryotic sequences (i.e., AGS4; Fig. 1), which we refer to as ‘free-living’ prokaryotic communities unless otherwise indicated.

### Prokaryote genome sizes increase with ocean depth and lifestyle

**Fig. 2:**
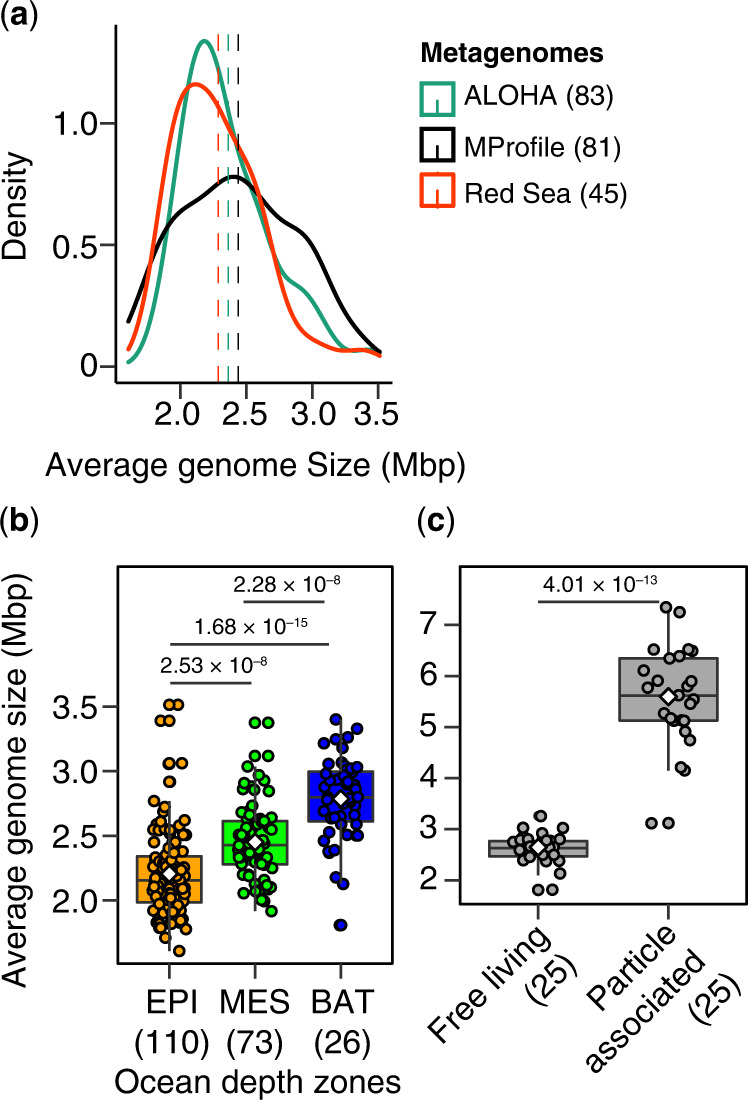
Distribution of genome size of uncultured marine prokaryotes. **a** Density distribution plots of estimated average genome size (AGS) of “free-living” marine microbial communities (0.1–3 µm) in three separate large-scale metagenomic collections of the pelagic to bathypelagic (up to 4000 mbsl) microbiome. The dashed lines show the mean AGS estimate for each metagenomic collection. The distributions are all consistent with unimodality [one-tailed, Hartigan’s *dip* test for unimodality, *p* = 0.567 (KRSE2011), 0.8194 (ALOHA), and 0.918 (MProfile)]. **b** AGS patterns correlate with ocean depth zones in global microbial metagenomes (*n* = 209) that includes prokaryotes in the epipelagic (EPI, ~3–200 m), mesopelagic (MES, > 200–1000 m), and bathypelagic (BAT, > 1000–4000 m). **c** AGS estimates in the “free-living” (0.2–0.8 µm) and particle-associated (0.8–20 µm) bathypelagic microbiome sampled latitudinally at 4000 m depth during the Malaspina expedition. Boxplots show the median as middle horizontal line and interquartile ranges as boxes (whiskers extend no further than 1.5 times the interquartile ranges). Data are shown as circular symbols, while mean values are shown as white colored diamonds. Values at the top indicate the adjusted significant *P*-values of the unpaired (**b**) and paired (**c**) two-sided Wilcoxon test with Benjamini-Hochberg correction. The number of metagenomes analyzed is indicated in parentheses in all three panels. Source data are provided as a Source Data file.

In three independent microbial metagenomic collections (Supplementary Data 1) comprising temporal (ALOHA, *n* = 83)^8^, regional (Red Sea, *n* = 45)^33^, and global (Malaspina Vertical Profiles, MProfile, *n* = 81)^36^ ocean microbiome surveys, we found distributions of AGS estimates that were consistently unimodal (Hartigan’s *dip* test for unimodality, *p* = 0.567–0.918) in the ‘free-living’ prokaryotic communities (0.1–3 µm) sampled from the surface (3 m) to the bathypelagic ocean (~4000 m; Fig. 2a). Notably, the Malaspina Vertical Profile metagenomes (Supplementary Data 1) covered a much wider range of ocean depths (3 to 4000 m) and latitudes than previous studies estimating AGS from oceanic metagenomes, but with relatively few samples^8,13,16^, providing a unique resource for our objectives. Combined analysis of all three metagenome datasets (Red Sea, ALOHA, and MProfile) revealed significant differences (Kruskal-Wallis χ^2^ = 72.762, df = 2, *p* < 2.2 × 10^–16^) in AGS estimates of marine prokaryotes inhabiting the three canonical ocean depth layers (Fig. 2b). Epipelagic prokaryotes (~3–200 m) had mean (± SD) and median AGS estimates of 2.21 ± 0.33 and 2.15 Mbp, respectively (*n* = 110). Mean (± SD) and median AGS estimates continued to increase for mesopelagic prokaryotes (> 200–1000 m) with values of 2.45 ± 0.29 and 2.43 Mbp, respectively (*n* = 73), and were highest for bathypelagic prokaryotes (> 1000–4000 m) with values of 2.92 ± 0.27 and 2.95 Mbp, respectively (*n* = 26).

The median AGS estimate range of 2.2 to ~3.0 Mbp in the sampled free-living (0.1–3 µm in size) marine prokaryotic communities (*n* = 209 metagenomes) is consistent with other large-scale metagenome sequence-based estimates and the sizes of metagenome-assembled prokaryotic genomes (MAGs; in 0.22–3 µm filters) from the photic ocean (surface to mesopelagic) based on the *Tara* Oceans Expedition (1.5–2.3 Mbp)^15,16^. Overall, our metagenome sequence-based AGS estimates support the unimodal distribution of prokaryotic genome sizes recently demonstrated in environmental genomes in several biomes^38^ and on cultured isolates (including marine bacterioplankton)^14,39^. However, estimates from isolates are likely biased since current cultivation approaches tend to favor copiotrophs (see, ref. ^3^).

We next tested whether the derived AGS estimates depended on microbial cell size by analyzing 25 paired bathypelagic metagenomes (MDeep; Supplementary Data 1) sampled during the global Malaspina Expedition^40^ in which both prokaryotic life strategies, free-living (0.2–0.8 µm size) and particle-associated (0.8–20 µm size), were sampled simultaneously^35^. The analyzed metagenomes (MDeep) were from the Atlantic, Pacific, and Indian Ocean provinces and cover a spatial distance of 9437 km with an average depth (± SD) of 3688 ± 526 m at the tropical and subtropical latitudes (–33.55°N to 32.0788°N). These microbial metagenomes were also screened for contaminating eukaryotic and viral sequences as indicated in Fig. 1a (see details in the “Methods” section and Supplementary Data 1). The genomes of bathypelagic prokaryotes associated with marine particles (5.6 ± 0.97 Mbp) were twice as large (paired two-sided Wilcoxon test, *p* < 0.0001) as those of their free-living counterparts (2.65 ± 0.3 Mbp; Fig. 2c). Crucially, these AGS estimates are also consistent with read-based predictions from bathypelagic waters (4000 m) in the Pacific Ocean (Station ALOHA)^13^ and estimates based on MAGs (~3.6 Mbp) recently compiled from both cell size fractions of the same bathypelagic metagenomes^35^.

The significant increase in AGS estimates with depth (Fig. 2b) and the twofold larger AGS for particle-associated compared to free-living bathypelagic prokaryotes (Fig. 2c) suggest larger genome size patterns in the hadal biosphere. Extending our analysis to metagenomes spanning hadal to abyssal depths (4000–10,500 m) based on seven recent Pacific Ocean metagenomes sampled from the Challenger Deep of the Mariana Trench^41,42^ yielded AGS estimates in the range of 3.46–4.19 Mbp and 3.88–4.92 Mbp for free-living (0.2–3 µm) and particle-associated (> 3 µm) prokaryotes, respectively (Supplementary Data 3). These estimates are also consistent with those of MAGs reconstructed from the same metagenomes in the Challenger Deep (Mariana Trench)^43^. Overall, this reinforces the patterns of larger AGS in particle-associated compared to free-living bathypelagic prokaryotes, and larger microbial genomes in the deep ocean compared to the upper ocean.

### AGS patterns are not geographically constrained

Examination of the geographic patterns of AGS estimates showed that AGS distribution was independent of geographic distance in both the regional (Red Sea, Mantel statistic *r* = 0.01824, *p* = 0.2971) and global (MProfile, *r* = –0.01413, *p* = 0.7924) ocean metagenomes. Furthermore, AGS estimates in the vertically profiled global Malaspina metagenomes (MProfile, *n* = 81) were significantly independent of the Longhurst biogeochemical province sampled (*n* = 9; Kruskal-Wallis χ^2^ = 1.0006, df = 8, *p* = 0.9982; Supplementary Data 1). The lack of covariance between the patterns of AGS estimates and geographic distance or Longhurst province sampled may reflect the high connectivity of microbial communities throughout the global ocean, particularly the redistributive effects of circulation by ocean currents and other transport processes, as well as the enormous population sizes of plankton that allow dispersal constraints to be overcome^44,45^. This is consistent with the relatively small differences in microbial assemblages recently found in different ocean basins^23,46^. Another possible explanation is the effect of seasonality, which can cause selection of different taxa, resulting in the succession of microbial communities and affecting their distribution (see, ref. ^47^), and thus influence AGS patterns.

An assessment of the relationship between AGS and measured environmental variables (Supplementary Fig. S1; Data 1)—separately for the Red Sea metagenomes (regional scale) and Malaspina Vertical Profiles metagenomes (global scale), showed that the cumulative effect of temperature, salinity, dissolved oxygen, and depth on AGS patterns was significant at both the regional scale (*n* = 45; Mantel statistic *r* = 0.1944, *p* = 0.0057) and the global scale (*n* = 81; Mantel statistic *r* = 0.1779, *p* = 1 × 10^–4^). This result suggests that environmental conditions are a driving force behind predicted AGS patterns in the marine microbiome. While no significant interaction effect was evident between many environmental variables (i.e., salinity, depth, oxygen, nitrate, and phosphate) in controlling AGS patterns (one-way ANOVA, *p* < 0.05; Supplementary Data 4), we found that depth and temperature covaried significantly, as expected (Spearman’s *r* = –0.72; *p* = 1.3 × 10^–14^).

Further statistical tests of the relative importance of environmental factors in linear regression analyses based on variance decomposition^48^ showed that temperature and depth (and nitrate in the case of the Red Sea) had similar but higher relative importance as predictors of AGS patterns than salinity or dissolved oxygen (Supplementary Fig. S2). The strong relationship between temperature and depth is consistent with evidence for a strong temperature dependence of pelagic bacterioplankton diversity and composition from single-cell genomics and metagenomics^3,23^. However, our results extend this assessment from the photic ocean studied previously to the deep ocean interior, where the maximum depth of the studied samples (4000 m; Supplementary Fig. S1) reflects the average global ocean depth^20^. This in turn captures the extensive microbiome of uncultured marine microorganisms in the dark and cold deep ocean, the largest habitat by volume in the ocean^20^. Overall, these results suggest strong environmental selection on microbial genome sizes, with comparatively little constraint on the dispersal of the prokaryotic community of average genome size in the global ocean.

### Scaling laws predict microbial genome sizes in the thermally stratified ocean

**Fig. 3:**
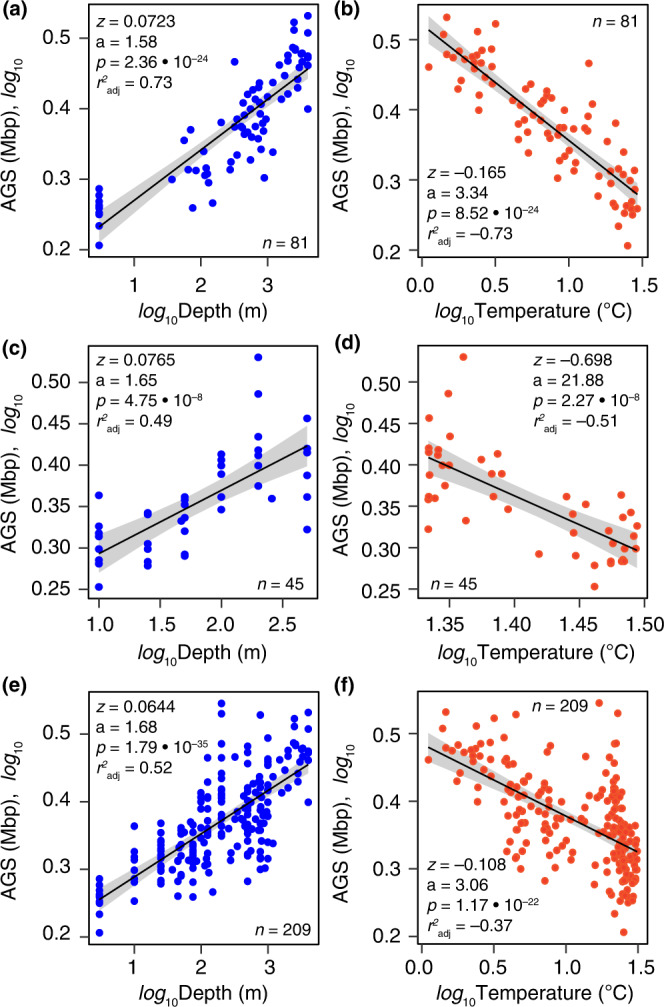
Scaling rate of genome size of marine prokaryotes with abiotic factors. A linear fit on a logarithmic scale illustrates the power law rates at which estimated average genome size (AGS) changes with depth (**a**, **c**, **e**) and temperature (**b**, **d**, **f**) for metagenomes at global (Malaspina Profile, **a**, **b**) and regional (Red Sea, **c**, **d**) scales. Panels **e** and **f** show the same scaling relationships in these two datasets, but analyzed together with the temporal dataset from Station ALOHA (*n* = 83). The solid black line and grey error bands (**a**–**f**) indicate the regression curve and 95% confidence intervals for the best power law curve fit, respectively. The power law exponent (*z*), intercept (a), model significance *p*-value (*p*), and the adjusted coefficient (*r*^2^_adj_) are shown in individual panels based on the *F*-test. Further details are provided in Supplementary Data 5 and 6. Source data are provided as a Source Data file.

Based on the above findings, we used the gathered data at global (MProfile), regional (Red Sea), and temporal (ALOHA) scales and performed curve-fitting analysis for each data set individually and for all three together to find the best regression models that accurately explained the relationship between the AGS estimates and depth or temperature. We found that power laws best represented the relationship between AGS estimates and these two variables (Fig. 3; Supplementary Data 5). AGS estimates increased with a power of 0.072 of depth (Fig. 3a) and decreased with a power of –0.165 of temperature (Fig. 3b) on the global scale (Malaspina). Examination of these correlations for a local Red Sea data set exhibiting extreme surface temperatures (up to 32 °C; Supplementary Fig. S1; Data 1) and remarkably uniform but high temperature (~22 °C) from 200 m to the seafloor (2000–3000 m)^49^, effectively reproduced the patterns observed at the global scale (Fig. 3c, d; Supplementary Data 5). Although the samples spanned a one-month expedition^33^, which was not possible during the global Malaspina expedition (from December 2010 to July 2011)^34–36^, this consistency of results increases confidence in the observed global trends and suggests that the inferred relationships are robust predictors of the underlying ecological factors. However, a steeper rate of AGS change with temperature was observed in the regional dataset (*z* = –0.698; *n* = 45; Fig. 3d) than in the global dataset (*z* = –0.165; *n* = 81; Fig. 3b), suggesting that AGS changes more rapidly with temperature extremes.

Next, we examined the effect of seasonality on our scaling predictions using a temporally resolved, localised dataset from the subtropical North Pacific ocean (Station ALOHA), where metagenomes were collected over 10 months (August 2010 to December 2011)^8^. Similar scaling relationships of AGS with depth and temperature were also found (Supplementary Fig. S3; Data 5), reinforcing patterns from the regional and global datasets (Fig. 3). However, linear regression analyses applied to individual sampling months (seasons) revealed greater variance in the direction of the relationship between AGS and environmental factors (Supplementary Fig. S4–S7), suggesting that seasonal dynamics may affect AGS patterns in marine prokaryotes. For example, correlation analyses revealed significant (Spearman *r* = –0.71 to 0.90; *p* < 0.05) but opposing relationships in the spring, summer, and winter seasons (Supplementary Fig. S4–S7). As expected, the scaling exponent of the rate of change of AGS with temperature (*z* = –0.07) and the regression strength (*p* ≤ 0.001) were much lower when all temporal metagenomes (*n* = 83) were analyzed together (Supplementary Fig. S3) or analyzed together with the previous datasets (Fig. 3e, f; Supplementary Data 6), compared to the separate results from the regional (Red Sea) and global (Malaspina) datasets (Fig. 3a–d; Supplementary Data 5). In contrast, the scaling exponent of the rate of change of AGS with depth remained relatively unchanged for all three comparisons (Fig. 3a, c, e). Overall, these results support the robustness of the scaling laws predicting the rate of change of AGS with depth and temperature in the global ocean microbiome (Fig. 3), despite the inferred seasonal effects.

### Rates of change of AGS with respect to temperature and depth are different in photic and aphotic tropical oceans

Seawater column temperature profiles vary at different latitudes; surface water is warmer near the equator and colder at the poles. In low-latitude tropical regions, the sea surface is much warmer, resulting in a highly pronounced thermocline. In addition, the surface temperature changes relatively little seasonally in tropical regions (hence there is little seasonal change in the profiles). At high latitudes (polar regions), there is little difference between surface temperature and temperature at depth, and temperature is fairly constant (and cold) at all depths regardless of season. Thus, if the predicted scaling factor between AGS and temperature or depth is universal, we expect that (1) horizontal transects at a given depth spanning a wide range of temperatures should have the same scaling relationship with AGS in the tropic ocean and that (2) perennially cold polar marine waters should have the same linear relationship between AGS and depth.

**Fig. 4:**
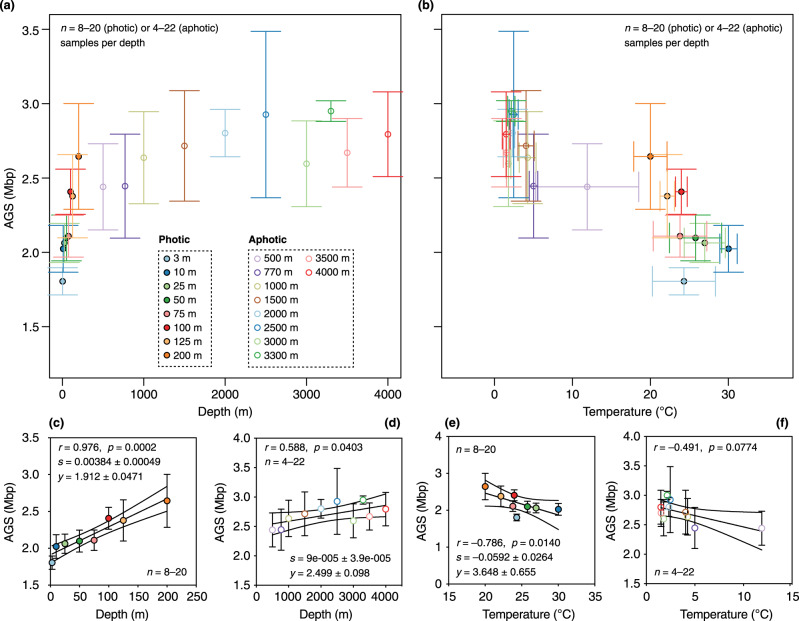
The rate of change of AGS estimates with depth and temperature is greater in the thermocline. Two-way plots of mean (circular symbols) of the estimated average genome size (AGS) and depth (**a**) and between AGS estimates and temperature (**b**) across horizontal transects at similar ocean depths at the global scale (*n* = 204 metagenomes). Horizontal and vertical error bars depict standard deviation of the mean depth (x-axis in panel a) or temperature (x-axis in **b**) and the mean AGS [y-axis in panels (**a**) and (**b**)]. Only metagenomes represented by four independent samples at similar depths in different sampling locations were considered (*n* = 4–22 metagenomes per depth). Circular symbols are color-coded by depth, with closed and open circles denoting samples from the photic (3–200 m, 8 depths; *n* = 106, 8–20 metagenomes per depth) and aphotic (> 200–4000 m, 10 depths; *n* = 106, 4–22 metagenomes per depth) layers, respectively. **c**–**f** Spearman rank correlations (one-tailed) between mean AGS estimates and depth (**c**, **d**) or temperature (**e**, **f**) in the photic (**c**, **e**; *n* = 8–20 metagenomes per depth) and aphotic (**d**, **f**; *n* = 4–22 metagenomes per depth) layers. The circular symbols show the mean AGS estimate and the error bars show the standard deviation (SD). The solid black line and grey dashed lines indicate the regression curve and 95% confidence intervals for the curve, respectively. The means (± SD) of regression slope (*s*) and y-axi*s* intercept (*y*) are given for curves that were significantly linear (*p* < 0.05). Source data are provided as a Source Data file.

Accordingly, we examined the relationship between AGS and depth or temperature in horizontal transects at similar depths in ocean metagenomes sampled worldwide (*n* = 204; Supplementary Data 1). We found that the relationship between AGS and these two variables (conditioned on the same horizontal depth) clearly separated the photic epipelagic ocean (3 to 200 m; *n* = 8 depths; Fig. 4a) from the aphotic ocean (mesopelagic and bathypelagic; *n* = 10 depths; Fig. 4b). In both cases, the dependence of AGS on depth (Fig. 4c) and temperature (Fig. 4d) was significantly linear (*p* < 0.05; Fig. 4c, e) only in the photic layer, but not in the aphotic layer (Fig. 4d, f). This again suggests that the genome size landscape of uncultured marine prokaryotes in the global ocean is clearly separated along the dimensions of the two abiotic factors. However, the estimated regression slope of AGS with depth (mean ± SD: 0.0038 ± 0.00049; Fig. 4c) was lower than that for temperature (mean ± SD: −0.0591 ± 0.0264; Fig. 4e), suggesting that the rate of change of AGS with temperature is 16-fold higher than with depth. This corresponds to a change of approximately 4 and 59 genes per unit of depth and temperature, respectively, assuming an average-sized microbial protein-coding gene of 1000 base pairs in planktonic prokaryotes found at similar depths up to 200 m. The observed distinct pattern of change in AGS with these environmental variables in photic and aphotic oceans likely reflects the pronounced thermocline conditions in the photic layer at low latitudes.

**Fig. 5:**
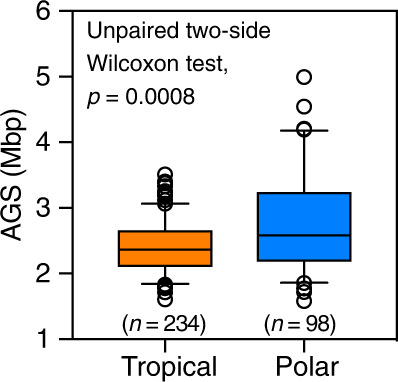
Marine prokaryotic genomes are larger in the polar ocean than in the tropical low-latitude ocean. Boxplots show estimated median average genome size (AGS) as horizontal lines and interquartile ranges as boxes (whiskers extend to a maximum of 1.5 times the interquartile range). Mean values are shown as white colored diamonds. Values at the top indicate the adjusted significant *P* values of the unpaired two-sided Wilcoxon test with Benjamini-Hochberg correction. Values in parenthesis show the number of metagenomes. Source data are provided as a Source Data file.

Next, we examined the relationship between AGS and both variables in polar metagenomes (*n* = 94) extending from the surface to 3800 m depth (Supplementary Data 1) and covering different transects in the Arctic and Antarctic Oceans^50^ and the *Tara* Ocean Polar Circle Expedition^51^. Unlike tropical waters at low latitudes, polar waters do not have a thermocline and temperatures do not exhibit pronounced seasonal variations. This raises the question of whether the same inverse relationship between AGS and temperature exists in permanently cold polar marine waters. However, we found no statistically significant linear correlation (*p* < 0.05) between AGS and depth or temperature (Supplementary Fig. S8), suggesting that AGS varies invariably in permanently cold waters regardless of seawater depth. To gain further insight, we compared AGS estimates between metagenomes from the tropical ocean (*n* = 234) and those from high-latitude polar waters (*n* = 98), both extending from the surface to the bathypelagic ocean. The results show that polar waters contain free-living prokaryotes (0.1–3 µm) with significantly larger genomes (unpaired two-sided Wilcoxon test, *p* = 0.0008) than their counterparts from low-latitude tropical oceans (mean ± SD: 2.73 ± 0.72 Mbp vs. 2.41 ± 0.39 Mbp; Fig. 5). These results reinforce our earlier finding that prokaryotic genomes from low-latitude tropical marine waters are larger in the cold interior deep ocean than in the photic ocean (see above), and they corroborate reports of large genomes in metagenome-resolved genomes from the polar Arctic^51^. However, the findings leave unanswered the question of whether interior ocean prokaryotes harboring large genomes have a greater metabolic capacity due to additional genes in the genome.

### Gene lengths and family sizes correlate strongly with microbial genome sizes

If the smaller AGS estimate in prokaryotic communities occupying the photic ocean are due to fewer gene duplications and shorter coding genes in the streamlined bacteria of the surface ocean^3,8^, then we expect that the potential for gene family expansion (duplications) and elongation of coding genes predominates in microorganisms with larger genomes colonizing the nutrient-rich interior of the ocean. Therefore, we examined the relationship between AGS and coding gene traits, including the average gene length (AGL), percent GC content, and gene family size, using full-length coding genes previously assembled for global Malaspina Vertical Profiles metagenomes (i.e., MProfile)^34^. We defined gene families as the number of unique protein-coding genes distinct to each metagenome regardless of water column depth, based on a catalog of 32.7 million full-length non-redundant genes reconstructed from the same metagenomes^34^. The gene catalog contains microbial coding gene sequences (effectively protein families) clustered at 95% global sequence identity and 80% overlap across the shorter gene. The processed matrix of gene family copies across all MProfile metagenomes (available here, 10.6084/m9.figshare.19673688.v1) was used to compile a binary table of gene presence and absence and the corresponding counts of unique genes per metagenome (Supplementary Data 1).

**Fig. 6:**
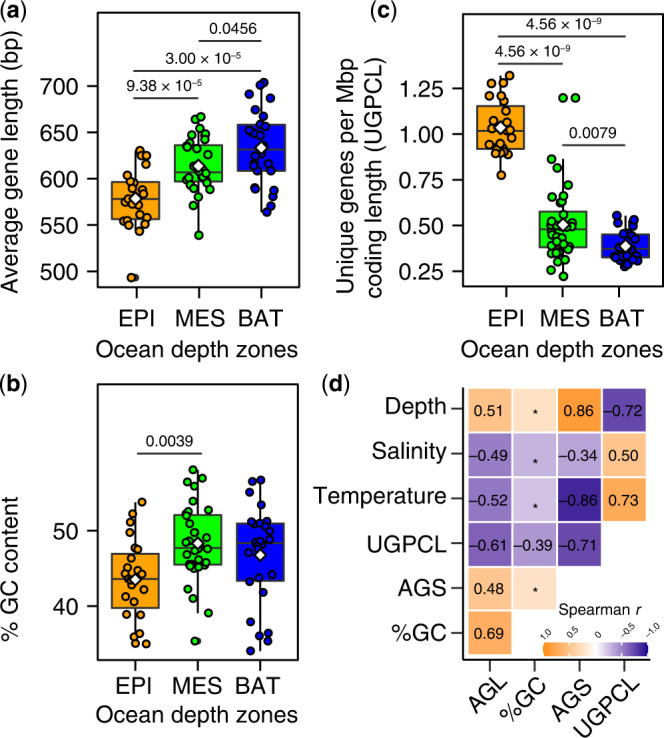
Larger marine prokaryotic genomes harbor elongated and highly redundant coding gene sequences. Box-and-whisker plots (**a–c**) show estimated average gene length (AGL), percent guanine and cytosine (% GC), and number of unique genes per megabase pair (Mbp) of coding sequence length (UGPCL) in metagenomic assemblies from the global Malaspina expedition (*n* = 81). Depth and number of samples analyzed are as follows: the epipelagic (EPI, ~3–200 m; *n* = 23), mesopelagic (MES, > 200–1000 m; *n* = 32), a*n*d bathypelagic (BAT, > 1000–4000 m; *n* = 26). Boxplots show median as horizontal lines and interquartile ranges as boxes (whiskers extend to a maximum of 1.5 times the interquartile range). Mean values are shown as white colored diamonds. Values at the top indicate the adjusted significant *P*-values of the unpaired two-sided Wilcoxon test with Benjamini-Hochberg correction. **d** Relationship between genetic (AGL, %GC, and UGPCL) and environmental (temperature, depth, and salinity) factors in the sampled global ocean microbiomes (*n* = 81). Values indicate Spearman correlations coefficient (one-tailed). Asterisks mark non-significant relationships (*p* > 0.01). Source data are provided as a Source Data file.

Both AGL (Fig. 6a) and GC content (Fig. 6b) increased with ocean depth, whereas the number of unique genes per unit coding sequence length (Fig. 6c) or sequenced metagenomic effort (Supplementary Fig. S9) decreased with depth. The median AGL in the three traditional ocean depth layers were 583 bp (epipelagic), 612 bp (mesopelagic), and 637 bp (bathypelagic), indicating a relative length increase of 54 bp from the upper to bathypelagic ocean (Fig. 6a). At the same time, the number of unique genes per coding sequence length (UGPCL) decreased by a factor of two from the epipelagic to bathypelagic ocean (Fig. 6c). These results are consistent with reports of microbial gene length shortening in the low-nitrogen, sunlit ocean^3,8^ and gene duplications observed in microbial species with larger genomes, including representative marine prokaryotes^52,53^, and in environmental genomes of deep-sea microbes^13^.

Spearman rank correlation analyses also showed that the AGL of coding genes was significantly positively correlated with changes in AGS, GC content, and water column depth (*r* = 0.48–0.69, *p* = 10^−^^4^–10^−^^10^; Fig. 6d), but significantly negatively correlated with UGPCL and temperature (*r* = −0.49 to −0.61, *p* = 10^−^^6^–10^−^^9^; Fig. 6d). In contrast, UGPCL was significantly positively correlated with temperature (*r* = 0.73, *p* = 3.1 × 10^–21^), but negatively correlated with depth (*r* = −0.72, *p* = 4.8 × 10^−^^8^), AGL (*r* = −0.61, *p* = 3.3 × 10^−^^9^), GC content (*r* = −0.39, *p* = 0.0013), and AGS (*r* = −0.71, *p* = 1.2 × 10^−^^13^). Collectively, these results suggest that streamlining selection favors shorter coding genes in the photic ocean and that the size patterns of microbial genomes in the interior ocean reflect the elongation of coding genes and expansion of gene family size, which likely leads to genetic redundancy.

## Discussion

Previous studies aimed at understanding the patterns and ecological determinants of genome size in marine microbes have been limited to cultured microbial representatives with biased diversity^14^ and relatively few metagenome datasets from the photic zone and samples with limited geography and water column depth^8,13,15,16^. An understanding of genome evolution in marine prokaryotes must account for the greater diversity of uncultured microbial communities in the ocean and the environmental forces that likely constrain adaptation, including the ecologically relatively stable interior of the deep ocean. Using regional and global metagenomic analyses, our results show that the average genome size (AGS) of environmentally abundant and uncultured marine bacteria and archaea is smaller in the epipelagic ocean than in the deep ocean. Importantly, our estimates of AGS of uncultured marine prokaryotes are consistent with previous predictions and metagenome-resolved genomes independently assembled in different ocean provinces^8,13,15,35,43^. This in turn, underscores the relative robustness of our approach to estimating genome size in metagenomes on a global scale.

The pelagic ocean is vertically structured by gradients of light, temperature, oxygen, and dissolved nutrients that strongly influence the genetic and functional repertoire of its microbial inhabitants^23^. In contrast, deep-sea microorganisms face a number of insurmountable challenges, including high hydrostatic pressure and cold temperatures. Previous reports suggested that the low GC content of prokaryotes in the photic zone may be an adaptation to nutrient deficiency^3,8^. Other studies suggested that nutrient deficiency is a driver of microbial genome size reduction in the pelagic oligotrophic ocean^12^ and that natural selection favors organisms with larger genomes under nutrient-rich conditions (e.g., in the deep ocean)^12^. Therefore, it is likely that the genomic landscape of prokaryotes in the global ocean, at both low and high latitudes, is ecologically differentially constrained in photic and aphotic marine environments. By examining the relationship between genome size and environmental variables, we confirm the observed increase in genome size with depth and temperature at regional scales^8,13^ and extend the temperature dependence of AGS patterns in marine microbiomes to the global ocean and up to the hadal biosphere. Our results identify depth and temperature as robust predictors of the microbial genome size landscape in the thermally stratified ocean.

Why does the genome size of prokaryotes in the ocean predictably and significantly correlate with temperature than depth? Several possibilities are conceivable. The most parsimonious explanation is the mutually reinforcing effect of temperature on the rate of biological processes (see, ref. ^54^) and spontaneous mutation rates^26,27^. Physiological rates generally increase two- to threefold when temperature increases by 10 °C^55^. Consequently, a greater fraction of reaction molecules are above activation energy at higher temperatures in the photic ocean than in the deep ocean interior, where temperatures are consistently three- to fourfold lower. This selectively favors small microbial genomes in the sunlit pelagic ocean, which have a compact gene inventory as ocean surface temperatures approach the theoretical optimum for growth (see, ref. ^56^), and may significantly reduce the metabolic cost of DNA replication and the efficiency of gene expression in shorter operons in pelagic phytoplankton compared to the large genomes of dark ocean microorganisms. Remarkably, marine bacterioplankton species such as *Pelagibacter* (Alphaproteobacteria) and *Prochlorococcus* (Cyanobacteria), which have compact genomes, also have large global population sizes on the order of 10^26^ to 10^28^ cells^57^, highlighting the ecological importance of genome reduction in the sunlit pelagic ocean.

Moreover, the temperature dependence of spontaneous mutation rates [see, ref. ^26,27^] can accelerate mutation rates much more in the warm upper ocean than in the dark interior ocean. Also, given the persistence of ultraviolet-induced DNA damage in the photic ocean^58^, maintaining genome integrity comes at a significant energy cost and can lead to mutations^59^. Consequently, prokaryotic populations, such as the ecologically abundant epipelagic bacteria *Prochlorococcus* and *Pelagibacter*, are subject to much stronger purifying selection than those in the dark ocean, as reflected by their higher mutation rates^10,60,61^. Interestingly, higher mutation rates are viewed as the main driver of genome size reduction in prokaryotes^5,7^, but this view is also changing, as recent studies implicate genetic drift as a key driver of genome evolution [see, ref. ^62^].

We also find that gene duplication—either via paralogs, horizontal gene transfer, or both—is a selection mechanism that may have led to the proliferation of potentially large microbial genomes in the nutrient-rich and cold interior of the ocean. This is consistent with reports of high gene duplication in bacteria with large genomes^63,64^ and the demonstrated high rates of gene gain in the mesopelagic microbiome through horizontal gene transfer^65^, which is considered an adaptation to cold water^65^. Given that low temperatures generally slow mRNA translation^66,67^ and that gene redundancy reduces the number of translations of a gene by multiple copies of the same gene, suggests that increased gene dosage might help overcome temperature limitations on metabolic rates in the cold ocean interior. For example, through rapid protein synthesis and cell division to facilitate adaptation, making gene duplication a potentially important ecological strategy in the cold deep ocean. Alternatively, gene duplication and size increase (e.g., for protein stability and enhanced catalysis) could be a safeguard for enhancing gene expression to exploit abundant nutrients in the deep sea at low temperatures. Remarkably, both species and functional richness increase with water column depth, while cell density and potential maximum growth rate decrease with depth^23,68^. Thus, the increase in genome size in the deep, dark ocean is also associated with slower biomass turnover^68,69^, which is reflected in the much longer turnover times of heterotrophic microbes in the interior ocean below 200 m (~0.8 years) than in the upper ocean (6–25 days)^69^.

Overall, our results demonstrate the temperature and depth dependence of genome size in native marine prokaryotic communities. The patterns observed in the global ocean (this study), in biofilm bacterial communities in rivers across latitudinal gradients^70^, and in diverse soils^71^ suggest that the selection of small microbial genomes at high temperatures is a nearly universal ecological trend in natural ecosystems. We argue that the relaxation of temperature constraints on metabolic rates favors lean biochemical networks and high mutation rates in warm pelagic waters of the tropical ocean, and consequently small microbial genomes. Importantly, we found that the rate of change of AGS was exceptionally stronger in the epipelagic ocean (up to 200 m) than in the aphotic ocean (i.e., mesopelagic and bathypelagic), especially with respect to temperature than depth. This trend was reinforced by the steep rates of AGS change with temperature in the Red Sea regional dataset, where temperatures are extreme at the surface (32 °C) and remain constant at 22 °C from 200 m to the seafloor. This suggests that the size of microbial genomes is strongly influenced by thermocline conditions in the ocean. Ultimately, this has implications for the generation time and evolution of microbial genomes in the photic ocean as the oceans continue to warm due to global climate change.

The large repertoire of accessory (unique) genes associated with small microbial genomes in the upper ocean provides an arsenal of functions to cope with the changing pelagic environment and is likely a major reason for the large population sizes of native pelagic prokaryotes (i.e., *Pelagibacter* and *Prochlorococcus*). Of evolutionary significance, these pelagic prokaryotes, which exhibit extreme genome reduction, also possess unique islands of genome variability acquired through horizontal gene transfer that facilitate adaptation to local environments and provide viral defense^72,73^. In contrast, we find that the enlargement of deep-sea prokaryote genomes through gene duplication is an important evolutionary force in the interior ocean, likely playing a role in niche adaptation and possibly reflect greater metabolic versatility in resource utilization [see, ref. ^35^], lower purifying selection as suggested elsewhere^13^, or complex abiotic and biotic interactions. Given that most of the ocean volume lies below the photic zone (where low temperatures and high pressures dominate)^20^, the finding that larger genomes prevail in the dark ocean has implications for the genetic and biodiversity landscape in the ocean, where microbial diversity is presumed greater at depth than in the photic layer (see, ref. ^23^), but which remain largely unexplored.

## Methods

### Metagenomic datasets

Four metagenomic collections were used in this study based on recent compilations by Duarte et al.^34^. These include two datasets from the Malaspina 2010 Expedition^40^, namely Malaspina Vertical Profiles (MProfile; *n* = 81; BioProject No. PRJEB52452)^36^ and Malaspina deep-ocean metagenomes (MDeep; *n* = 50; European Nucleotide Archive (ENA) No. PRJEB44456)^35^. Also, included were the localized metagenomes from the Red Sea (KRSE2011; *n* = 45; BioProject No. PRJNA289734)^33^ and temporal datasets from the North Pacific Subtropical Gyre (Station ALOHA; *n* = 83; BioProject No. PRJNA352737)^8^. Unless otherwise noted, only metagenome collections where two or more depths were sampled per station were used for downstream analyses. Therefore, the final metagenomes used (Supplementary Data 1) do not necessarily reflect the original counts in Duarte et al. ^34^. For example, the analyzed MProfile metagenomes contain 81 samples (out of 100) from the same stations covering multiple depths. For the station ALOHA, only 83 metagenomes (out of 116) were used; these also contained environmental variables used for correlation analysis.

Additional datasets covering the bathypelagic domain (MDeep)^35^ and the hadal biosphere (Yap Trench and Mariana Trench)^41,42^ were analyzed independently of the vertically sampled metagenomes (MProfile, Station ALOHA, and KRSE2011). Specifically, 50 MDeep metagenomes (out of 58)^35^, with matched ‘free-living’ and particle-associated fractions were considered. Trench metagenomes were retrieved from the NCBI Short Reads Archive database under BioProject No. PRJNA479337 and PRJNA412741 with accession numbers SRP151902 (Yap Trench) and SRP119520 (Challenger Deep Mariana Trench), respectively.

Finally, polar ocean metagenomes were also obtained from ENA based on the recent studies by Royo-Llonch and colleagues^51^, who sampled marine waters in the Polar Arctic Circle (*n* = 34; BioProject PRJEB9740), and Cao and colleagues^50^, who sampled various marine transects in the Arctic and Antarctic Oceans (*n* = 60; BioProject PRJNA588686). Of note, temperature data are only available from the *Tara* Ocean Polar Arctic Expedition^51^, but not from the second study, where only depth was provided^50^. Therefore, different sample counts (out of 98) are used for the correlation analysis of AGS with respect to temperature (*n* = 34) and depth (*n* = 94). These metagenomes are also listed in Supplementary Data 1.

All metagenomes were subjected to quality control as described in Duarte et al. ^34^. Briefly, the raw metagenome sequences were quality filtered and trimmed using Trimmomatic (v0.39)^74^, followed by removal of PhiX sequencing control reads using BBMap v38.90^75^. The quality-checked paired-end reads were then error corrected using the workflow “Tadpole.sh” implemented in BBMAP with the options “cc=t rollback=t pincer=t tail=t prefilter=t prealloc=t mode=correct”. The quality of the sequenced reads in all these steps was assessed using FASTQC^76^. The high-quality metagenome reads were used for downstream applications following the same procedures as shown in Fig. 1a, including: (1) independent assembly using metaSPAdes v3.15.2^77^ with preset metagenomic options and a Kmer range of 21 to 127; (2) retention of contigs with a minimum size of ≥ 500 bp; and (3) de novo removal of “contaminating” eukaryotic and viral reads based on corresponding assemblies (≥ 500 bp contigs), prior to average genome size (AGS) determination with MicrobeCensus v1.1.1^22^. MicrobeCensus aligns metagenomic reads against a set of thirty universally conserved single-copy marker genes found in bacteria and archaea to rapidly and accurately estimate AGS. Default settings were used, except that the number of reads (option “-n”) for AGS estimation was set to 10 million. Full details can be found below. Based on the workflow shown in Fig. 1a, the AGS estimate of uncultured marine prokaryotes was predicted using the preprocessed high-quality metagenomic sequences (Supplementary Data 1).

### Estimating average genome size in marine prokaryotes using metagenomic reads

Given the likely impact of viral and eukaryotic reads, which include small- and large-genome-sized organisms on the predictions of AGS, we first performed three independent prescreening steps to remove these “contaminants” in the metagenomic reads. Accordingly, four separate AGS estimations (designated as AGS1 to AGS4) were inferred to examine the effects of ‘contaminating’ reads—from small eukaryotes, their gametes, or putative viruses included in the surveyed ocean microbiomes, on the derived AGS estimates. The goal of these analyses was to obtain high-quality sequences that did not include putative eukaryotic and viral metagenomic sequences that would likely bias AGS estimates.

AGS1 is derived directly from the preprocessed high-quality metagenomic reads that we assumed contained these contaminants. For AGS2 to AGS4, we iteratively screened the preprocessed high-quality metagenomic reads for potential eukaryotic and viral read sequences prior to AGS estimation. Because no current genome database captures the entirety of uncultured eukaryotic and viral genomes present in the ocean, we leverage two de novo approaches to retrieve genome sequences of eukaryotic and viral DNA sequences from assembled metagenomic contigs. Individual metagenomes were assembled independently using metaSPAdes v3.13.1^77^ in the metagenome mode. The draft metagenome assemblies were then filtered by contig size to retain sequences with a minimum length of 500 bp, followed by de novo screening of potential eukaryotic and viral contig sequences using EukRep v0.6.2^78^ and VIBRANT v1.2.1^79^, respectively. EukRep was run with the options “--m strict --min 1000”, while VIBRANT was run with a minimum contig length of 1 kbp.

Contig sequences that were flagged as putative eukaryotes were then used as queries to remove the corresponding eukaryotic reads contaminating the respective metagenomes using BBMap v38.22 (https://github.com/BioInfoTools/BBMap/) by mapping the high-quality metagenomic reads against these contaminating sequences. The resulting unmapped reads were used to calculate AGS2 with MicrobeCensus (as described above), which is equivalent to AGS predictions without environmental eukaryotic genomes. A second mapping of the above “eukaryotes-free” reads was performed using BBMap separately against viral genomes in RefSeq (accessed October 8, 2020) and contigs predicted de novo as putative viruses with VIBRANT. After each of these steps, the unmapped reads were retained to calculate AGS3 and AGS4 estimates using MicrobeCensus, which correspond to AGS predictions without viral genomes from RefSeq (AGS3) and those predicted de novo (AGS4), respectively. Importantly, AGS4 provides a robust estimate of genome size exclusively from prokaryotes in marine metagenomes in the absence of eukaryotic and viral genomes.

A one-way ANOVA (repeated-measures analysis) was performed to determine whether putative eukaryotic and viral sequence affected AGS estimations in unassembled metagenomes, suggesting procedural bias. Overall, the results indicate that AGS estimates derived directly from preprocessed metagenomes (AGS1) were significantly higher than those in which eukaryotic (AGS2) and viral (AGS3) reads were removed de novo (Supplementary Data 2). Thus, unless otherwise stated, AGS results reported in the main text are based on AGS4 predictions—that is, on high-quality metagenomes without read sequences of the two contaminants (Supplementary Data 1). AGS4 was subsequently used to perform various statistical tests comparing AGS estimates in ‘free-living’ versus particle-associated marine prokaryotes, correlates of geographic distance, environmental variables, and genetic traits.

### Statistical analyses

Statistical analyses were carried out in R v4.0.1 (www.R-project.org). The following R packages were used for the specified analyses as described below, with plots created using “ggplot2” v3.3.3^80^.

Base R was used for linear regression. Significant differences were tested using one-way analysis of variance (ANOVA) or repeated measures ANOVA using the package “rstatix” v0.6.0^81^. False discovery rates (α = 0.05) were corrected for multiple comparisons based on the Benjamini-Hochberg correction method. Data were normally distributed as deduced from the Shapiro-Wilk normality test. Distributions that violated statistical assumptions were analyzed using nonparametric tests (i.e., Spearman correlation and Mann-Whitney U test).

Simple (nonparametric) correlation analyses were calculated from the correlation matrix of the response variables and plotted using “ggcorrplot” v0.1.3^82^. Mantel tests were performed to correlate AGS patterns with geographical distances or environmental factors using “vegan” v2.5–7^83^. Geographical distances reflect Haversine distances between two points estimated with “geosphere” v1.5–10 ^84^ based on sampled latitude and longitude coordinates.

The frequency distribution of AGS estimates, which deviate from unimodality, was tested with the Hartigan’s dip test statistic (HDS) using “dip test” v0.75-7^85^. Simulated p-values based on 500 bootstrap replicates were used for the test. HDS essentially tests the statistical significance that a distribution can be divided into two or more distinct parts. AGS estimates in metagenomes with *p*-values (> 0.01) were categorized as significantly unimodal.

A sample-specific linear correlation analysis of AGS estimates from ALOHA with environmental variables was calculated using “easystats” v0.4.3^86^. Curve fitting for the selection of non-linear regression models (i.e., power, logist, and exponential models) was conducted using “REAT” v3.0.2^87^. The best model was selected on the basis of the *F*-test statistics [Pr (>F)], the probability of rejecting the null hypothesis, where one model does not fit significantly better (α = 0.05) than the model with zero slope.

A relative-importance analysis test was used to quantify the relative contributions of environmental variables (e.g., temperature, depth, salinity, and oxygen) to the changes in AGS patterns. The analysis was conducted using “relaimpo” v2.2–3^48^ with 500 bootstraps using three commonly used multiple-linear regression models (i.e., lmg, first, and last) from the methods provided by the relaimpo package. These methods allow estimating the contributions of explanatory variables in a multi-linear-regression model.

### Reporting summary

Further information on research design is available in the Nature Portfolio Reporting Summary linked to this article.

## Supplementary information

Supplementary Information

Description of Additional Supplementary Files

Supplementary Data 1–6

Reporting Summary

## Data Availability

All metagenomic data sets are publicly available in the European Nucleotide Archive (ENA) portal (https://www.ebi.ac.uk/ena/browser/home), the NCBI Short Reads Archive (https://www.ncbi.nlm.nih.gov/), or both. Accession numbers (PRJEB44456, PRJEB52452, PRJNA289734, PRJNA352737, PRJEB9740, PRJNA479337, PRJNA412741, and PRJNA588686) and sample identifiers and locations for the raw metagenomes are listed in Supplementary Data 1. Additionally, we made available the matrix of gene copies (10.6084/m9.figshare.19673688.v1) across individual samples of the Malaspina Vertical Profiles metagenomes. Source data are provided with this paper.

## Source data

Source Data
