# Supplementary material for: Abiotic selection of microbial genome size in the global ocean
Source: Nat Commun. 2023 Mar 13;14:1384. doi: 10.1038/s41467-023-36988-x (PMC10011403; doi:10.1038/s41467-023-36988-x)
Supplement: Supplementary file 1 — Supplementary Information [file 41467_2023_36988_MOESM1_ESM.pdf]

Abiotic selection of microbial genome size in the global ocean

David K. Ngugi[1*], Silvia G. Acinas[2], Pablo Sánchez[2], Josep M. Gasol[2], Susana Agusti[3], David M. Karl[4], and Carlos M. Duarte[3]

[1] Leibniz Institute DSMZ – German Collection of Microorganisms and Cell Cultures, Braunschweig, Germany; [2] Department of Marine Biology and Oceanography, Institut de Ciències del Mar, CSIC, Barcelona, Spain; [3] King Abdullah University of Science and Technology, Red Sea Research Center, Thuwal, Saudi Arabia; [4] Department of Oceanography, School of Ocean and Earth Science and Technology, University of Hawaií at Mãnoa, Honolulu, USA.

* Correspondence to: david.ngugi@dsmz.de

Supplementary Figures

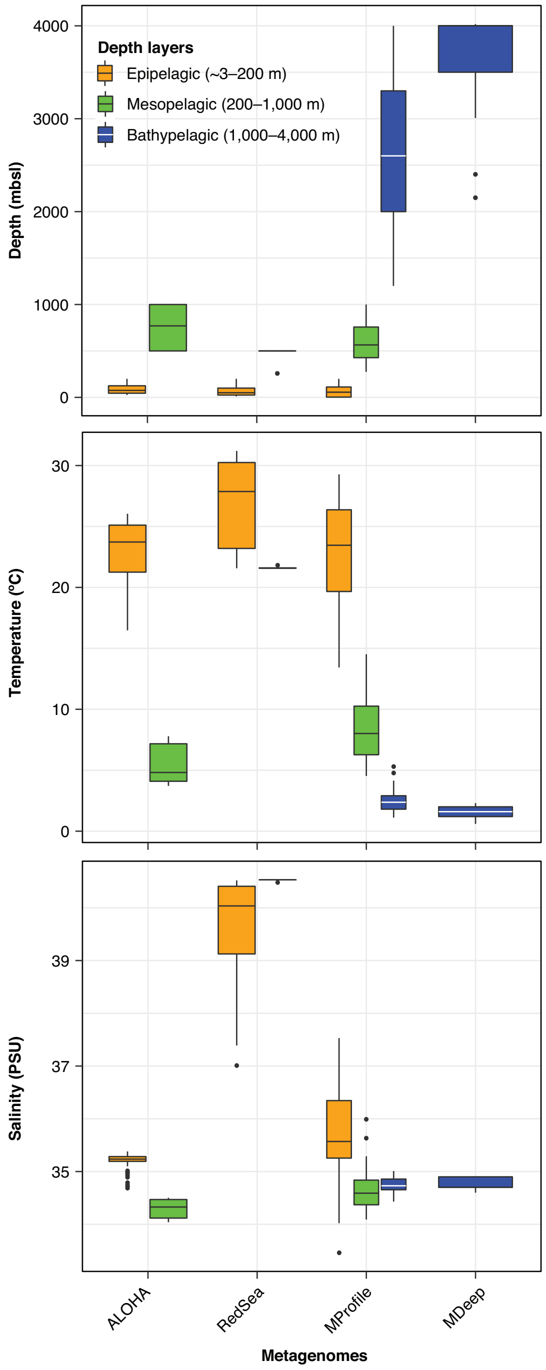

Fig. S1. **Extensive variation of environmental factors in the sampled ocean metagenomes**. Only depth, temperature, and salinity are shown because these co-occurred in all the metagenomes under this study. Additional data can be found in Supplementary Data 1. Boxplots show the median as middle horizontal lines and interquartile ranges as boxes (whiskers extend no further than 1.5 times the interquartile range). Depths of the three ocean zones are provided in parenthesis. The greatest depths were sampled in the Malaspina metagenomes (MProfile, n = 81 and MDeep, n = 50), while the highest temperature and salinity were measured in the epipelagic, especially in the Red Sea (n = 45). ALOHA, n = 83. The total number of metagenomes between the different ocean layers (epipelagic, mesopelagic, and bathypelagic) are respectively: ALOHA (EPI, 49;

MES, 34), Red Sea (EPI, 38; BAT, 7), Mprofile (EPI, 23; MES, 32; BAT, 26), and MDeep (BAT, 25). Source data are provided as a Source Data file.

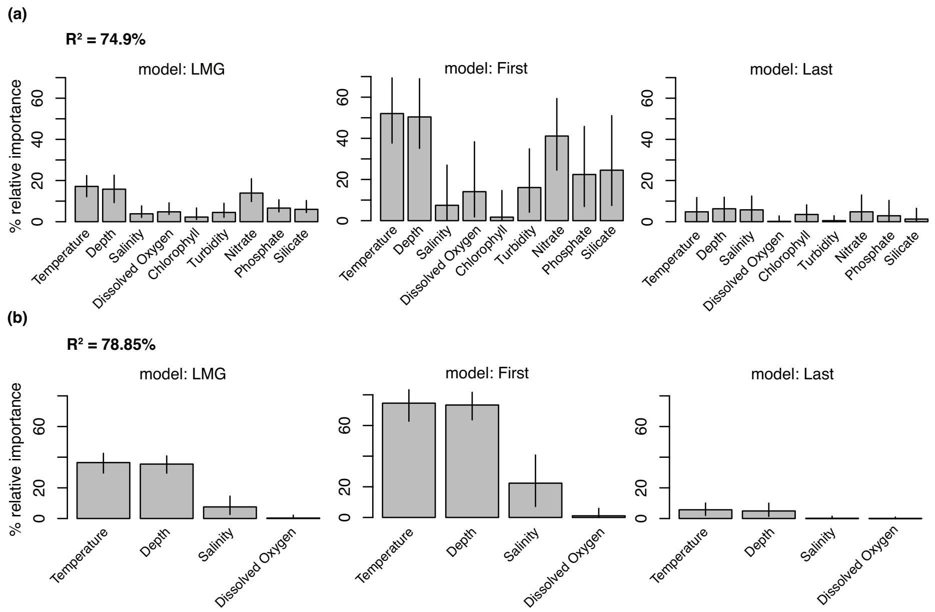

Fig. S2. **Temperature and depth are significant predictors of AGS patterns in the ocean microbiome**. Relative importance of environmental variables as predictors of estimated average genome size (AGS) patterns at regional (a) and global (b) levels based on metagenomes from the Red Sea (*n* = 45) and Malaspina expedition (*n* = 81), respectively. Analyses were performed using the R package "relaimpo" v2.2-3 [48] for univariate linear models with three regression models (LMG, First, and Last) and 500 bootstraps for confidence estimates. R^2 denotes the proportion of response variance explained by the model. The Lindeman-Melinda-Gold model (LMG) is the contribution of ordered predictors to R^2. In the model "First", each variable contributes when included first (the squared covariance between AGS and the variable), while in the model "Last", each variable contributes when included last (also sometimes called usefulness). Bars show the mean proportion of response variance (i.e., relative contribution) of each variable based on 500 bootstrap replicates. In panel "a" and "b", *n* = 45 and 81 values per variable, respectively. Error bars indicate the 95% bootstrap confidence intervals of the response variance (in percentage). All three models consider temperature and depth as the most significant contributors for the variance in AGS patterns. Source data are provided as a Source Data file.

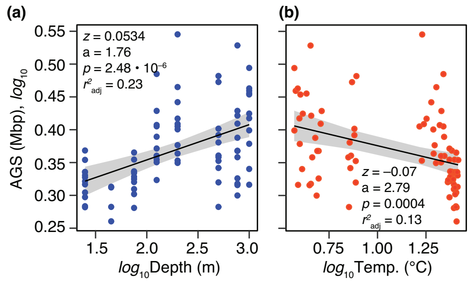

Fig. S3. **AGS scales lineally with temperature and depth at Station ALOHA**. Rate of AGS change also scale linearly with depth (a) and temperature (b) in matched metagenomes temporally sampled from station ALOHA (n = 83). Additional details are provided in Supplementary Data 5. The solid black line and grey error bands (in panels a and b) indicate the regression curve and 95% confidence intervals for the best power law curve fit, respectively. The power law exponent (z), intercept (a), model significance p-value (p), and the adjusted coefficient ($r^2{}_{adj}$) are shown in individual panels based on the F-test. Further details are provided in Supplementary Data S5. Source data are provided as a Source Data file.

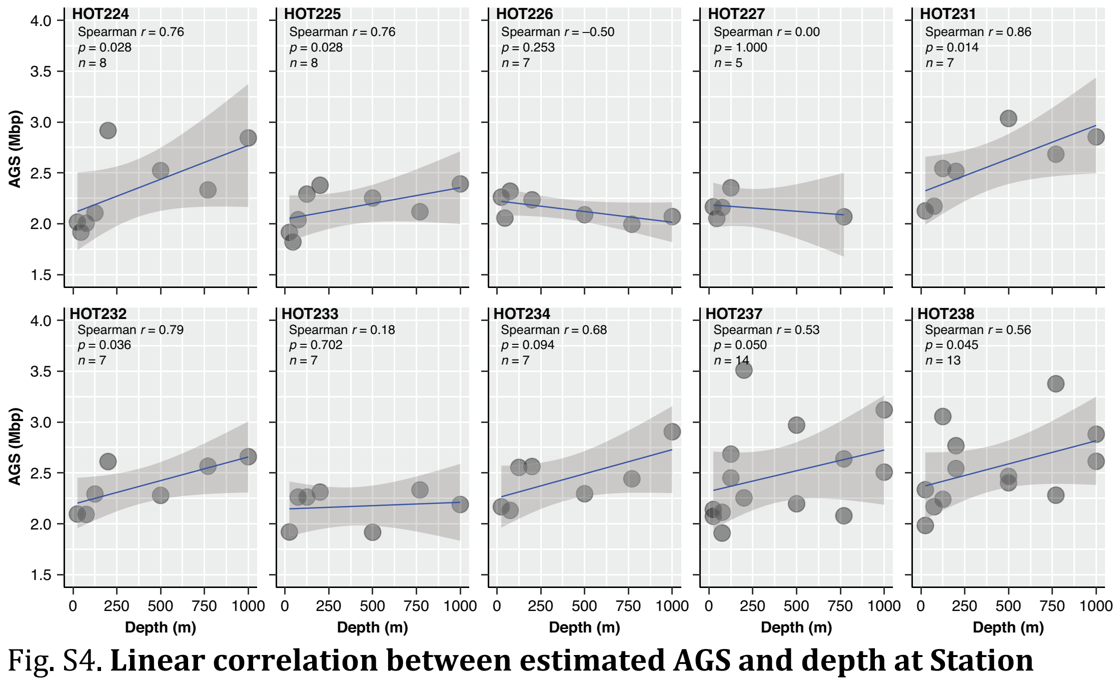

Fig. S4. **Linear correlation between estimated AGS and depth at Station ALOHA**. Spearman correlation of AGS estimates and depth for each metagenome collections ordered by sampling month (season) in station ALOHA as follows: August 2010, HOT224; September 2010, HOT225; October 2010, HOT226; November 2010, HOT227; April 2011, HOT231; May 2011, HOT232; July 2011, HOT233; August 2011, HOT234; November 2011, HOT237; December 2011, HOT238). The blue solid line shows the fitted linear curve (one-tailed), with the corresponding 95% confidence interval shown in grey shading. The number of metagenomes is shown in each panel and ranges from 5 to 13. Additional details are provided in Supplementary Data 1. Source data are provided as a Source Data file.

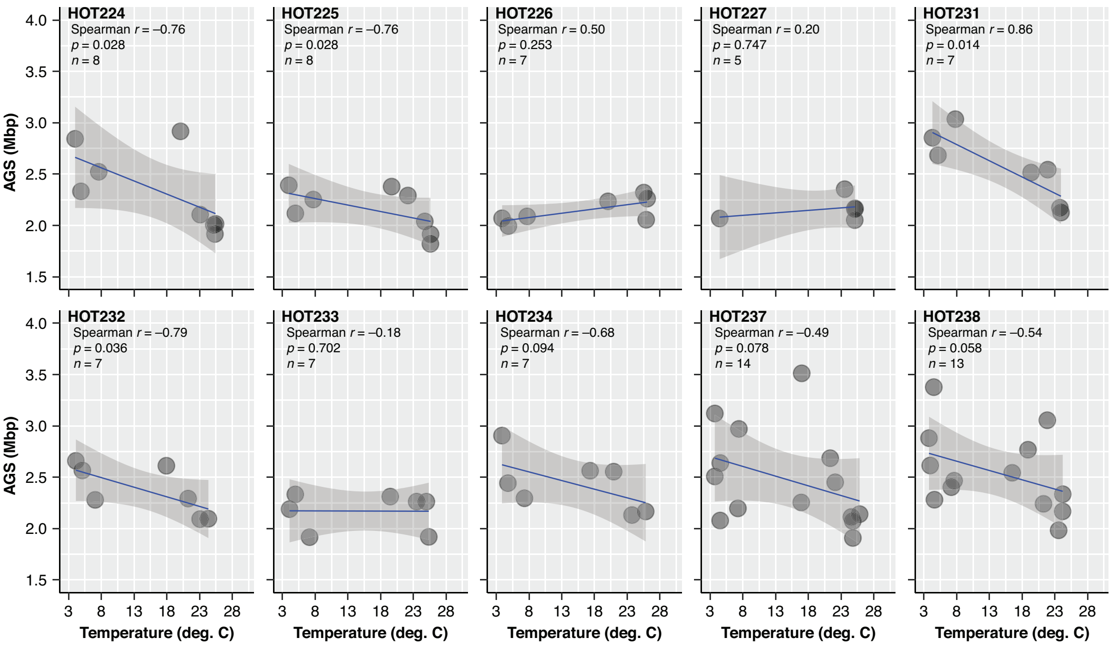

Fig. S5. **Linear correlation between estimated AGS and temperature at Station ALOHA**. Spearman correlation of estimated average genome size (AGS) and temperature for each metagenome collections ordered by sampling month (season) in station ALOHA as follows: August 2010, HOT224; September 2010, HOT225; October 2010, HOT226; November 2010, HOT227; April 2011, HOT231; May 2011, HOT232; July 2011, HOT233; August 2011, HOT234; November 2011, HOT237; December 2011, HOT238). The blue solid line shows the fitted linear curve (one-tailed), with the corresponding 95% confidence interval shown in grey shading. The number of metagenomes is shown in each panel and ranges from 5 to 13. Additional details are provided in Supplementary Data 1. Source data are provided as a Source Data file.

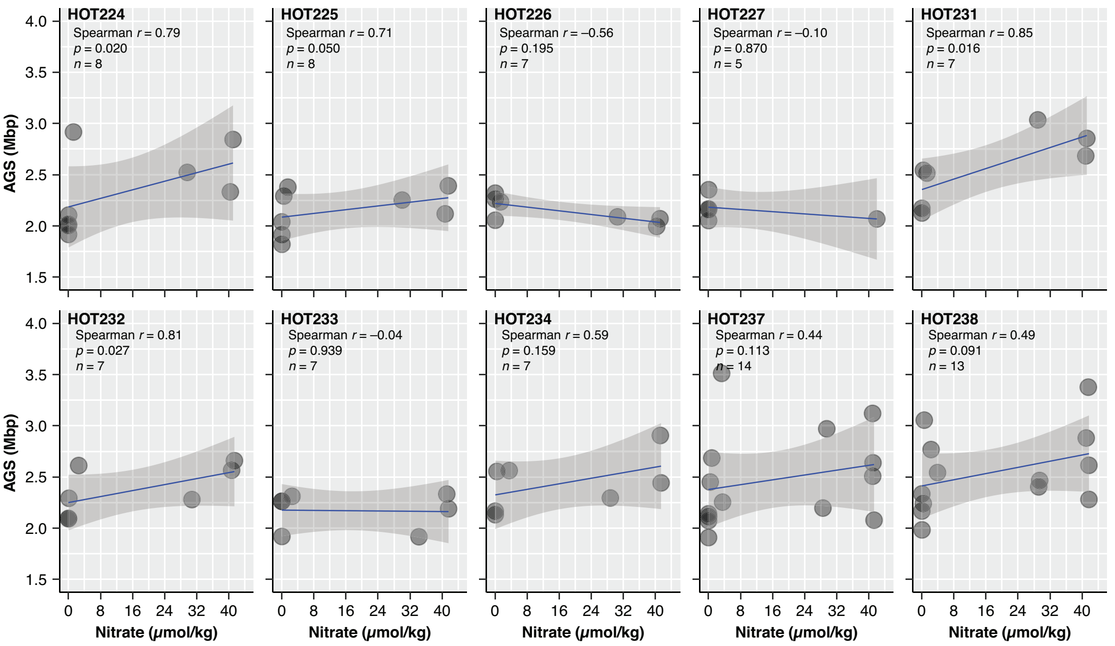

Fig. S6. **Linear correlation between estimated AGS and nitrate at Station ALOHA**. Spearman correlation of estimated average genome size (AGS) and nitrate for each metagenome collections ordered by sampling month (season) in station ALOHA as follows: August 2010, HOT224; September 2010, HOT225; October 2010, HOT226; November 2010, HOT227; April 2011, HOT231; May 2011, HOT232; July 2011, HOT233; August 2011, HOT234; November 2011, HOT237; December 2011, HOT238). The blue solid line shows the fitted linear curve (one-tailed), with the corresponding 95% confidence interval shown in grey shading. The number of metagenomes is shown in each panel and ranges from 5 to 13. Additional details are provided in Supplementary Data 1. Source data are provided as a Source Data file.

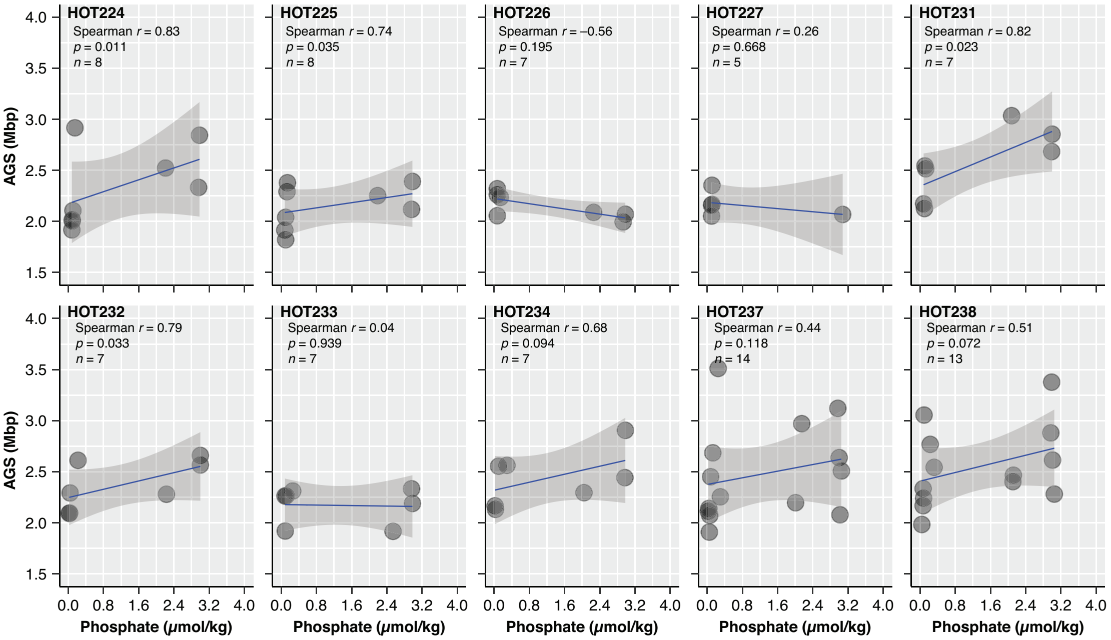

Fig. S7. **Linear correlation between estimated AGS and phosphorus at Station ALOHA**. Spearman correlation of estimated average genome size (AGS) and phosphorus for each metagenome collections ordered by sampling month (season) in station ALOHA as follows: August 2010, HOT224; September 2010, HOT225; October 2010, HOT226; November 2010, HOT227; April 2011, HOT231; May 2011, HOT232; July 2011, HOT233; August 2011, HOT234; November 2011, HOT237; December 2011, HOT238). The blue solid line shows the fitted linear curve (one-tailed), with the corresponding 95% confidence interval shown in grey shading. The number of metagenomes is shown in each panel and ranges from 5 to 13. Additional details are provided in Supplementary Data 1. Source data are provided as a Source Data file.

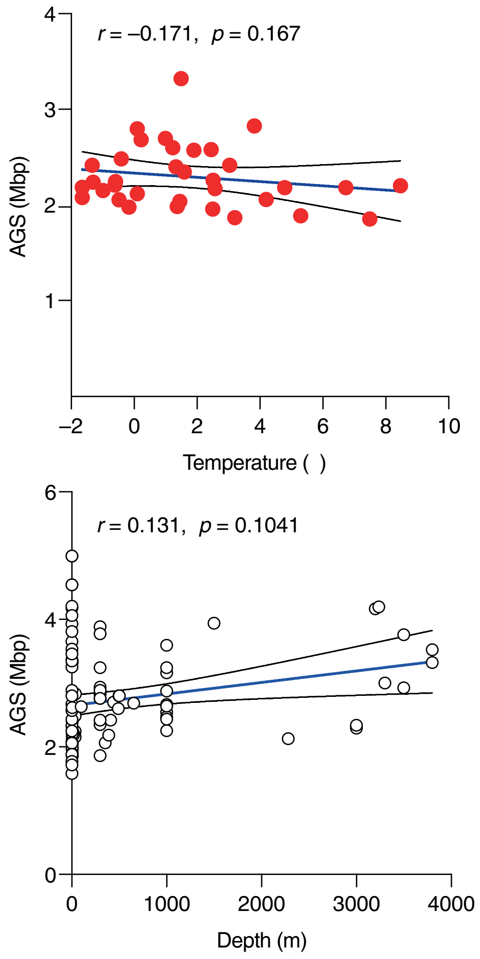

Fig. S8. **Linear correlation between estimated AGS and temperature or depth in the Polar Ocean.** Spearman rank correlations (*r*) between average genome size (AGS) estimates and temperature (**a**) or depth (**b**) in the polar ocean based on metagenomes from various transects in the Arctic and Antarctic Oceans [50] and the *Tara* Ocean Polar Circle expedition [51]. The number of metagenomes analyzed are respectively 34 (in panel a) and 94 (in panel b); four metagenomes without temperature or depth information were exclude from the correlation analysis. The blue solid line shows the fitted linear curve (one-tailed), with the corresponding 95% confidence interval denoted by the upper and lower black lines. However, none of the variables was significantly linear ($p > 0.05$) with AGS estimates. Source data are provided as a Source Data file.

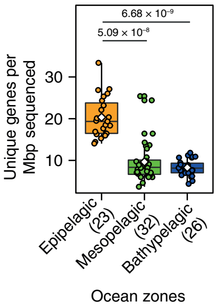

Fig. S9. **Gene redundancy increases with ocean depth**. Comparison of the number of unique genes per unit metagenome sequenced along different ocean layers based on the global Malaspina profile dataset (n = 81). Boxplots show the median as middle horizontal line and interquartile ranges as boxes (whiskers extend no further than 1.5 times the interquartile ranges). Data are shown as circular symbols, while mean values are shown as white colored diamonds. Values at the top indicate the adjusted significant P values of the unpaired two-sided Wilcoxon test with Benjamini-Hochberg correction. Values in parenthesis show the number of metagenomes in each depth zone. Source data are provided as a Source Data file.
