# Supplementary material for: Abiotic selection of microbial genome size in the global ocean
Source: Nat Commun. 2023 Mar 13;14:1384. doi: 10.1038/s41467-023-36988-x (PMC10011403; doi:10.1038/s41467-023-36988-x)
Supplement: Supplementary file 2 — Description of Additional Supplementary Files [file 41467_2023_36988_MOESM2_ESM.pdf]

Description of Additional Supplementary Files

File Name: Supplementary Data 1.
Description: General information about the metagenome collections together with the read counts and corresponding predicted AGS estimates after different read-processing methods (see workflow in Fig. 1).

File Name: Supplementary Data 2.
Description: Comparison of AGS estimates in different samples using repeated measures of one-way ANOVA before and after removal of "contaminating" eukaryotic and viral sequences in different metagenome collections.

File Name: Supplementary Data 3.
Description: Estimates of average genome size from trench metagenomes (Pacific Ocean) comprising “free-living” and particulate prokaryotic communities.

File Name: Supplementary Data 4.
Description: Analysis of variance (ANOVA) for linear regression between AGS estimates and multiple environmental factors in different metagenome collections.

File Name: Supplementary Data 5.
Description: Curve fitting for comparison of linear, power law, exponential, and logistic models of AGS estimates with depth and temperature in three metagenome collections, including the Red Sea (KRSE2011), the North Pacific Subtropical Gyre (ALOHA), the global Malaspina Expedition (MProfile).

File Name: Supplementary Data 6.
Description: Curve fitting for comparison of linear, power law, exponential, and logistic models of AGS estimates with depth and temperature based on AGS predictions of different metagenomes.
