# Supplementary material for: Abiotic selection of microbial genome size in the global ocean
Source: Nat Commun. 2023 Mar 13;14:1384. doi: 10.1038/s41467-023-36988-x (PMC10011403; doi:10.1038/s41467-023-36988-x)
Supplement: Supplementary file 4 — Reporting Summary [file 41467_2023_36988_MOESM4_ESM.pdf]

nature portfolio

Corresponding author(s): David K. Ngugi

Last updated by author(s): February 9, 2023

Reporting Summary

Nature Portfolio wishes to improve the reproducibility of the work that we publish. This form provides structure for consistency and transparency in reporting. For further information on Nature Portfolio policies, see our Editorial Policies and the Editorial Policy Checklist.

Statistics

For all statistical analyses, confirm that the following items are present in the figure legend, table legend, main text, or Methods section.

n/a	Confirmed	
☐	☒	The exact sample size (*n*) for each experimental group/condition, given as a discrete number and unit of measurement
☐	☒	A statement on whether measurements were taken from distinct samples or whether the same sample was measured repeatedly
☐	☒	The statistical test(s) used AND whether they are one- or two-sided<br>*Only common tests should be described solely by name; describe more complex techniques in the Methods section.*
☐	☒	A description of all covariates tested
☐	☒	A description of any assumptions or corrections, such as tests of normality and adjustment for multiple comparisons
☐	☒	A full description of the statistical parameters including central tendency (e.g. means) or other basic estimates (e.g. regression coefficient) AND variation (e.g. standard deviation) or associated estimates of uncertainty (e.g. confidence intervals)
☐	☒	For null hypothesis testing, the test statistic (e.g. *F*, *t*, *r*) with confidence intervals, effect sizes, degrees of freedom and *P* value noted<br>*Give P values as exact values whenever suitable.*
☒	☐	For Bayesian analysis, information on the choice of priors and Markov chain Monte Carlo settings
☒	☐	For hierarchical and complex designs, identification of the appropriate level for tests and full reporting of outcomes
☒	☐	Estimates of effect sizes (e.g. Cohen's *d*, Pearson's *r*), indicating how they were calculated

Our web collection on statistics for biologists contains articles on many of the points above.

Software and code

Policy information about availability of computer code

Data collection: No software was used for data collection

Data analysis: All software used for data analysis were open source and are described in details in the Methods section of the manuscript.
Below is a full list.
Metagenome sequence pre-processing:
Trimmomatic v0.39
BBMap Toolkit v38.90/ v38.22
FASTQC v0.11.8

Metagenome assembly, decontamination, and gene prediction:
metaSPAdes v3.13.1/ v3.15.2
VIBRANT v1.2.1
EukRep v0.6.2

Clustering of protein-coding gene sequences:
MMseqs2 v13.45111

Average genome size estimation:
MicrobeCensus v1.1.1

Statistical analyses and visualizations:
R v4.0.1 with packages:
ggplot2 v3.3.3
rstatix v0.6.0
easystats v0.4.3
REAT v3.0.2
dip test v0.75-7
relaimpo v2.2-3
ggcorrplot v0.1.3
vegan v2.5-7
geosphere v1.5-10

General data management:
Excel v2011

For manuscripts utilizing custom algorithms or software that are central to the research but not yet described in published literature, software must be made available to editors and reviewers. We strongly encourage code deposition in a community repository (e.g. GitHub). See the Nature Portfolio guidelines for submitting code & software for further information.

Data

Policy information about availability of data

All manuscripts must include a data availability statement. This statement should provide the following information, where applicable:

- Accession codes, unique identifiers, or web links for publicly available datasets
- A description of any restrictions on data availability
- For clinical datasets or third party data, please ensure that the statement adheres to our policy

All metagenomic datasets are publicly available at the European Nucleotide Archive (ENA) portal (https://www.ebi.ac.uk/ena/browser/home), the NCBI Short Reads Archive (https://www.ncbi.nlm.nih.gov/), or both. The accession numbers (PRJEB44456, PRJEB52452, PRJNA289734, PRJNA352737, PRJEB9740, PRJNA479337, PRJNA412741, and PRJNA588686) and sample designations and locations for the raw metagenomes are listed in Supplementary Table S1. In addition, we have provided the matrix of gene copies for each sample of the Malaspina Vertical Profiles metagenomes, which is available in FigShare (https://doi.org/10.6084/m9.figshare.19673688.v1).

Human research participants

Policy information about studies involving human research participants and Sex and Gender in Research.

Reporting on sex and gender	Does not apply.
Population characteristics	Does not apply.
Recruitment	Does not apply.
Ethics oversight	Does not apply.

Note that full information on the approval of the study protocol must also be provided in the manuscript.

Field-specific reporting

Please select the one below that is the best fit for your research. If you are not sure, read the appropriate sections before making your selection.

☐ Life sciences ☐ Behavioural & social sciences ☒ Ecological, evolutionary & environmental sciences

For a reference copy of the document with all sections, see nature.com/documents/nr-reporting-summary-flat.pdf

Ecological, evolutionary & environmental sciences study design

All studies must disclose on these points even when the disclosure is negative.

Study description	In this study, the average genome size (AGS) of free-living and particle-associated uncultured bacteria and archaea in marine waters was determined based on publicly available metagenomes, and the AGS estimates were correlated with environmental variables obtained simultaneously with the metagenome samples in tropical and polar oceans and up to the hadal region.
Research sample	The samples used in the study from publicly available metagenomes represent DNA sequences of seawater microbial communities captured in filters of 0.1–3 um in size. The DNA was sequenced using shotgun sequencing to retrieve the entire microbiome in a sample. For our study, the prokaryotic fraction in each DNA sequence (metagenome) was retrieved via dedicated software that

	removes eukaryotes and viral sequences in the pool of DNA sequences. The resulting bacterial and archaeal DNA sequences were the focus of the study.
Sampling strategy	All metagenome samples present in each publicly available BioProject (Accession numbers (PRJEB44456, PRJEB52452, PRJNA289734, PRJNA352737, PRJEB9740, PRJNA479337, PRJNA412741, and PRJNA588686) were retrieved. For every station/depth profile, we retained only samples were at least two depths were sampled. Sample sizes were independent of each dataset and no statistical tests were conducted to predetermine sample sizes.
Data collection	Publicly available sequence data from the European Nucleotide Archive (ENA) and NCBI Short Reads Archaive databases were collected for the study, including data from the Malaspina expedition, the Red Sea regional survey, temporal datasets from Station ALOHA (North Pacific Subtropical Gyre), the Tara Ocean polar circle, Antarctic and Arctic Ocean transects, and Yap and Mariana Deep.
Timing and spatial scale	Does not apply as the publicly available metagenomes were collected independently (different scales (regional, global and temporal), which served our purpose.
Data exclusions	No data were excluded except for the correlation analyses for Polar Ocean metagenomes, where no temperature or depth metadata was available for four metagenomes.
Reproducibility	All methods to reproduce the manuscript's results are described in detail in the manuscript. The metagenomes represent independent samples across different oceanic provinces.
Randomization	Does not apply as the metagenomes were sampled independently across different spatial-temporal scales.
Blinding	This study was not blinded as the metagenomes represent uncultured microbial entities present in the natural marine environment.

Did the study involve field work? ☐ Yes ☒ No

Reporting for specific materials, systems and methods

We require information from authors about some types of materials, experimental systems and methods used in many studies. Here, indicate whether each material, system or method listed is relevant to your study. If you are not sure if a list item applies to your research, read the appropriate section before selecting a response.

Materials & experimental systems

n/a	Involved in the study
☒	☐ Antibodies
☒	☐ Eukaryotic cell lines
☒	☐ Palaeontology and archaeology
☒	☐ Animals and other organisms
☒	☐ Clinical data
☒	☐ Dual use research of concern

Methods

n/a	Involved in the study
☒	☐ ChIP-seq
☒	☐ Flow cytometry
☒	☐ MRI-based neuroimaging
